# Analysis of Modified Plant Metabolites Using Widely Targeted Metabolite Modificomics

**DOI:** 10.21769/BioProtoc.5259

**Published:** 2025-04-05

**Authors:** Jianing Zhang, Shixuan Li, Yige Han, Shouchuang Wang, Penghui Liu, Jun Yang

**Affiliations:** 1 National Key Laboratory for Tropical Crop Breeding, School of Breeding and Multiplication (Sanya Institute of Breeding and Multiplication), Hainan University, Sanya, China; 2 National Key Laboratory for Tropical Crop Breeding, College of Tropical Agriculture and Forestry, Hainan University, Sanya, China; 3 Yazhouwan National Laboratory, Sanya, China

**Keywords:** Metabolomics, Modified metabolites, Q-Trap, QE-Orbitrap, Widely targeted

## Abstract

Metabolite modifications play a critical role in enhancing plants’ adaptability to environmental changes and serve as a major source of functional diversity in metabolites. However, current metabolomics approaches are limited to targeted analyses of a small number of known modified metabolites and lack comprehensive, large-scale studies of plant metabolite modifications. Here, we describe a widely targeted metabolite modificomics (WTMM) strategy, developed using ultra-high-performance liquid chromatography–quadrupole linear ion trap (UHPLC-Q-Trap) and ultra-high-performance liquid chromatography–Q-Exactive Orbitrap (UHPLC-QE-Orbitrap) technologies. This strategy enables high-throughput identification and sensitive quantification of modified metabolites. Using tomato as a model, we conducted a metabolite modificomics study and constructed a WTMM database, identifying 165 novel modified metabolites. The WTMM strategy is broadly applicable and can be extended to the study of other plant species.

Key features

• WTMM enables large-scale detection and quantitative analysis of plant-modified metabolites.

• Integration of UHPLC-Q-Trap and UHPLC-QE-Orbitrap technologies.

• The WTMM database is extensible and applicable to other plant species.

## Graphical overview


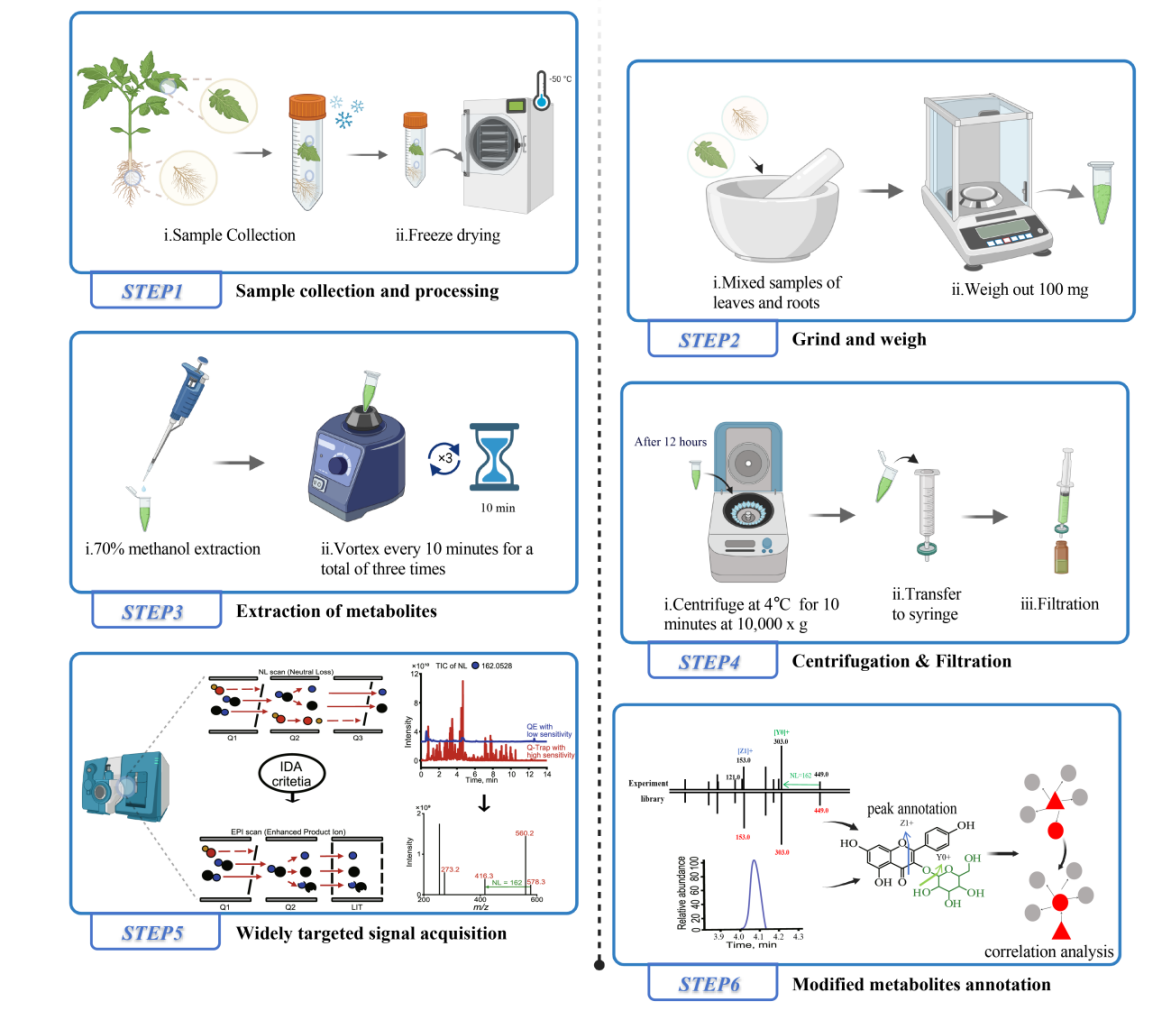


**Brief description of the tomato tissue extraction process for liquid chromatography–mass spectrometry (LC–MS) analysis and the widely targeted metabolite modificomics (WTMM) strategy.** The WTMM strategy employs an ultra-high-performance liquid chromatography–quadrupole-linear ion trap (UHPLC-Q-Trap) system in a stepwise neutral loss-enhanced product ion (NL-EPI) scanning mode to achieve high-throughput acquisition of modified metabolite profiles. Concurrently, high-resolution mass spectrometry data collected by the UHPLC-Q-Exactive-Orbitrap (UHPLC-QE-Orbitrap) system are utilized for structural annotation of metabolic signals. By applying the recursive algorithm of MetDNA, unknown metabolites (gray) are inferred based on correlation analysis with already identified metabolites (red). Created with BioRender.com.

## Background

Structural modifications of metabolites in plants play a pivotal role in various biological processes, including growth and development [1,2]. Different types of structural modifications endow metabolites with distinct chemical properties. For example, immature tomato fruits contain the toxic substance α-tomatine, which provides protection against predation. During fruit ripening, α-tomatine undergoes further acylation and glycosylation to produce the non-toxic substance esculeoside A, thereby enhancing the nutritional quality of the fruit [3]. These diverse types of modified metabolites constitute the metabolite modificomics, offering valuable insights into the diversity of plant metabolites.

There are many unknown types of structural modifications in plant metabolites, and current metabolomics techniques for detecting modified metabolites each have their own limitations and advantages. Nontargeted approaches allow for large-scale detection of metabolite signals but often lack the sensitivity required to detect ultra-trace levels of unknown modified metabolites [4]. Conversely, targeted methods are highly sensitive but can only detect known modifications, making it difficult to systematically study the full range of chemical modifications in plants [5]. The strategy presented in this study integrates the strengths of high sensitivity, broad coverage, and high resolution to enhance the detection sensitivity and identification efficiency for modified metabolites.

Here, we describe a widely targeted metabolite modificomics (WTMM) strategy [6], which employs the stepwise neutral loss-enhanced product ion (NL-IDA-EPI) method using ultra-high-performance liquid chromatography–quadrupole linear ion trap (UHPLC-Q-Trap) to capture metabolic signals containing various modified groups. High-resolution mass spectrometry (HRMS) data collected via ultra-high-performance liquid chromatography-Q-Exactive Orbitrap (UHPLC-QE-Orbitrap) are then used, alongside metabolite databases, for structural annotation of these signals, enabling the construction of a WTMM database. Furthermore, we utilize the scheduled multiple reaction monitoring (sMRM) mode of Q-Trap to perform quantitative analysis of the signals in the WTMM database, optimizing detection parameters to improve data quality. Using tomato as a model, we applied the WTMM strategy to establish a tomato-specific WTMM database, identifying 165 novel modified metabolites. Additionally, we demonstrated the utility of this database by validating diagnostic biomarkers for bacterial wilt in tomatoes [6].

## Materials and reagents

**Biological materials**

1. Tomato (MicroTom) seeds were surface sterilized at 50 °C for 30 min, germinated on moist filter paper in Petri dishes for two days, and then sown into trays filled with nursery soil. After five weeks, seedlings were transplanted into pots awaiting sampling or further treatment and planted at a density of 16.5 cm between plants in a row and 26 cm apart. Greenhouse management, including irrigation, fertilization, and pest control, essentially followed normal agricultural practices.

**Reagents**

1. Lidocaine (Sigma-Aldrich, catalog number: L1026)

2. Methanol (HPLC grade) (Merck, catalog number: 1.06035.2500)

3. Acetic acid (HPLC grade) (Merck, catalog number: 5.43808.0100)

4. Deionized water (Thermo Fisher Scientific, Lab Tower EDI 15 system)

5. Acetonitrile (HPLC grade) (Merck, catalog number: 1.00029.2500)

**Solutions**

1. 70% methanol extraction solvents (see Recipes)

2. Liquid chromatography solvent A (see Recipes)

3. Liquid chromatography solvent B (see Recipes)

**Recipes**

**1. 70% methanol extraction solvents (1,000 mL)**

**Table:** 

Reagent	Final concentration	Quantity or Volume
Methanol	n/a	700 mL
Deionized water	n/a	300 mL
Lidocaine	1 mg/L	1 mg

*Note: Store at 4 °C.*

**2. Liquid chromatography solvent A (1,000 mL)**

**Table:** 

Reagent	Final concentration	Quantity or Volume
Acetic acid	n/a	0.4 mL
Deionized water	n/a	1,000 mL

*Note: Prepare before use.*

**3. Liquid chromatography solvent B (1,000 mL)**

**Table:** 

Reagent	Final concentration	Quantity or Volume
Acetic acid	n/a	0.4 mL
Acetonitrile	n/a	1,000 mL

*Note: Prepare before use.*

**Laboratory supplies**

1. 150 μL glass inserts (Agilent, catalog number: 5183-2088)

2. 2 mL screw-top vials and caps (Agilent, catalog number: 5182-0716)

3. Captiva syringe filters, ValueLab 13 mm, 0.2 μm (Agilent, catalog number: 5190-5269)

4. 2 mL plastic centrifuge tube (Labshark, catalog number: 130201014)

5. Shim-pack GISS C18 column 2 × 150 mm, 5 μm (Shimadzu, catalog number: 227-30066-03)

6. 2 mL disposable syringes (Agilent, catalog number: 5610-2111)

## Equipment

1. Shimadzu Nexera X2 UHPLC system (Shimadzu, model: Nexera X2)

2. LC-MS/MS system (AB SCIEX, model: QTRAP 6500)

3. LC-MS/MS system (Thermo Fisher Scientific, model: Q Exactive Plus Orbitrap)

4. Mixer mill (Retsch, model: MM 400)

5. 1730R high-speed refrigerated microcentrifuge (Genespeed, catalog number: GS-1730R-NA)

6. MS 3 BASIC vortex oscillator (IKA, catalog number: 0003617000)

7. Freeze dryer (Christ, model: ALPHA 2-4 LD plus)

8. Ultra-low freezer, -80 °C (Sanyo, catalog number: MDF-U35V)

## Software and datasets

1. Analyst 1.6.3 (AB SCIEX, https://sciex.com)

2. Compound Discoverer 3.1 (Thermo Fisher Scientific)

3. MS2 Analyzer (http://fiehnlab.ucdavis.edu/projects/MS2Analyzer/)

4. MetDNA (http://metdna.zhulab.cn/)

5. Multi Quant 3.0.3 (AB SCIEX)

## Procedure

**A. Metabolite extraction steps**

**Figure 1.**
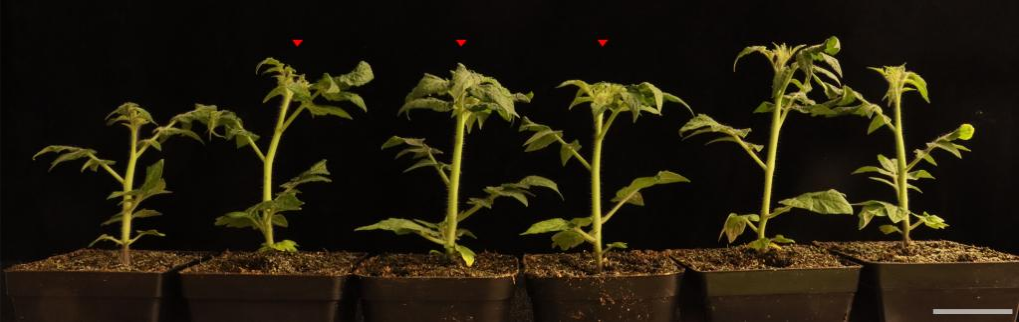
Selection of tomato plants. Randomly select three tomato plants with similar growth states to constitute one biological replicate.

**Figure 2.**
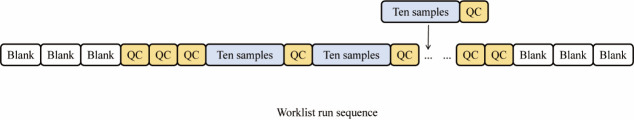
Sequence of worklist operations. Blank is used to eliminate background interference and check the status of the instrument; QC is to assess the stability and reproducibility of data during the mass spectrometry detection process.

1. Randomly select nine tomato plants with consistent growth (Figure 1). Group every three plants as one biological replicate and collect both leaves and roots into 50 mL centrifuge tubes with holes. Prepare a total of three biological replicates. After sample collection, immediately freeze the plants in liquid nitrogen. If freeze-drying cannot be performed immediately, the samples can be stored at -80 °C.

2. After 7 days of freeze-drying, the samples are ground using an MM400 grinder at a frequency of 25 Hz for 1.5 min. The ground tomato freeze-dried powder is stored at -80 °C. Scale bar, 5 cm.

3. Pre-chill the 70% methanol extraction solvent in a 4 °C refrigerator.

4. Weigh 0.1 g of freeze-dried tomato powder into a 2 mL centrifuge tube and add the prepared 70% methanol extraction solution to the tube at a ratio of 1 mL per 0.1 g. Seal and vortex the tube at maximum speed to ensure thorough mixing of the powder and solvent. After 10 min on ice, repeat the vortexing step for a total of three times. Afterward, place the tube in a 4 °C refrigerator and leave it to stand for 10 h.

5. After 10 h, vortex the centrifuge tubes containing the metabolite extraction solution again: Pre-cool the centrifuge to 4 °C and centrifuge the tubes at 10,000× *g* for 10 min. Carefully collect the supernatant and filter using a syringe with a 0.2 μm filter head. Transfer the filtered supernatant into injection vials and store them at -80 °C for subsequent LC–MS analysis of tomato metabolite samples. Before analysis, it is important to vortex the stored samples thoroughly to ensure proper mixing.

6. Quality control (QC) of the samples is necessary before analysis. The required control samples include a blank solution containing only methanol and a QC sample composed of equal volumes of all the samples. QC samples are used to assess the stability and reproducibility of the analysis and are periodically inserted during the analysis to monitor the process (Figure 2).

**B. LC method and parameters**

The liquid chromatograph we used is the Shimadzu Nexera X2. The chromatographic separation of compounds was achieved through a 15 min gradient elution in positive ion mode. During inverse-phase separation, the elution order of compounds is influenced by their polarity, with more polar compounds eluting first, while less polar components are more strongly retained on the column. Analytical conditions were as follows: column, shim-pack GISS C18 (pore size 5.0 μm, length 2 × 150 mm); mobile phase A, water (0.04% acetic acid) and mobile phase B, acetonitrile (0.04% acetic acid); temperature, 40 °C; and injection volume, 2 μL. The elution gradient for liquid chromatography is displayed in Table 1.

**Table 1.** Parameters for gradient elution in liquid chromatography.

Time (min)	Flow rate (mL/min)	Solvent A (%)	Solvent B (%)
0	0.4	95	5
10	0.4	5	95
11	0.4	5	95
11.1	0.4	95	5
15	0.4	95	5

**C. MS method and parameters**

**Figure 3.**
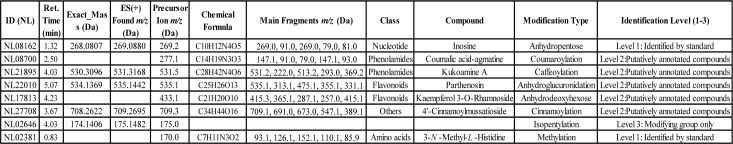
Template for the tomato widely targeted metabolite modificomics (WTMM) database. The data in the table are based on UHPLC-Q-Trap and UHPLC-QE-Orbitrap techniques combined with neutral loss scanning (NL) and high-resolution mass spectrometry (HRMS) analyses, which identified a variety of modification types including novel modification metabolites (e.g., parthenosin). Some metabolites (e.g., inosine) were directly verified by standards (Level 1) and the rest were annotated by fragmentation patterns and modification group characterization (Level 2/3). ID (NL), unique identifier of metabolite; Ret. Time (min), retention time of liquid chromatography; ES (+) Found. m/z (Da), molecular charge ratio of the detected molecules in the electrospray positive ion mode.

1. Use the NL-IDA-EPI mode of the API 6500 Q-Trap LC-MS/MS system to collect data on neutral losses in the tomato samples to ensure broad coverage of modified groups. The main parameters for the ESI source are as follows: ion source temperature, 500 °C; ion spray voltage (IS), 5500 V; ion source gases I (GSI), II (GSII), and curtain gas (CUR) set to 55, 60, and 25 psi, respectively; collision gas (CAD) set to high mode. The neutral loss value starts from 14 Da and takes 1 Da steps up to 245 Da. The precursor ion scanning range is set to 19–1,000 Da. In NL-IDA-EPI mode, Q1 scans all metabolic signals, fragments them in Q2 (collision cell) to produce product ions, and then scans these product ions in Q3. When Q3 detects a preset NL value (14–245 Da) with sufficient response intensity, information-dependent acquisition (IDA) is triggered to activate the linear ion trap module, and enhanced product ion scanning is performed to obtain MS2 spectra of precursor ions with neutral losses.

2. The precursor ion information (Q1) and product ion information (Q3) of the metabolic signals collected in NL-IDA-EPI mode, including all fragment ions (Q4, Q5, Q6, etc.), retention times (RTs), and the response intensities of precursor and product ions, are summarized. Duplicates with the same precursor ion, product ion, and retention time are removed, and signals with a signal-to-noise ratio (SNR) lower than 10 are discarded, resulting in a preliminary dataset of metabolic signals.

3. Data collection for tomato samples is performed on the Q Exactive Plus Orbitrap mass spectrometer in full MS/dd-MS2 mode, with a precursor ion scanning range of 50–1,000 Da. The main parameters for the ESI source are as follows: sheath gas, 40 psi; auxiliary gas, 12 psi; spray voltage, 3,000 V; capillary temperature, 360 °C; S-lens voltage, 55 V; and auxiliary gas heater temperature, 350 °C. The high-resolution metabolic signal data obtained are integrated with the Q-Trap data, and the precursor ion information (Q1), product ion information (Q3, including all fragment ions), and RTs are aligned and deduplicated. This results in the establishment of a high-quality tomato metabolite modificomics MS2T database [7], which includes high-resolution precursor ion Q1, product ion Q3, and RTs.

4. To facilitate the identification and annotation of metabolic signals, MS2Analyzer software is used to automatically identify and characterize neutral losses (NLs) in the MS2T dataset [8]. The m/z, RT, fragment information, and types of neutral losses for the metabolites obtained are compared with existing standard spectral databases for identification. For metabolites lacking commercial standards, commercial databases and literature searches are used to aid in their identification. High-resolution data are submitted to Compound Discoverer 3.1 software to match online databases for annotation of some metabolites. For other metabolites, manual identification is performed, and literature searches are conducted to compare metabolite information with those in the MS2T database for final identification.

5. The identified metabolite information is imported into MetDNA, and unknown metabolites are further identified through recursive algorithms based on metabolic reaction networks (MRN) [9], resulting in the construction of the tomato WTMM database. More details about the database can be obtained in the supporting information of our previous study [6] (Figure 3).

6. The metabolic signals in the WTMM database are further optimized by a transition selection and MS parameter optimization pathway. For example, for a precursor ion with m/z of 476.3 Da, two high-abundance product ions (Q3) are selected from the MS2T library, and two candidate ion pairs (476.3/335.1 and 476.3/304.1) are formed by combining with the parent ion. These ion pairs are quantitatively analyzed using the scheduled multiple reaction monitoring (sMRM) scanning mode on the QTRAP system. The declustering potential (DP) and collision energy (CE) are set to 10, 20, 30, 40, 50, and 60, orthogonally combined into 36 sets of test parameters. Finally, chromatographic peaks with poor peak shape, low signal intensity, low SNR, or overly high signal intensity and peak height are removed. High-quality chromatographic peaks are retained, and the corresponding detection parameters are recorded for subsequent analyses.

## Data analysis

**Neutral loss group annotation**

**Figure 4.**
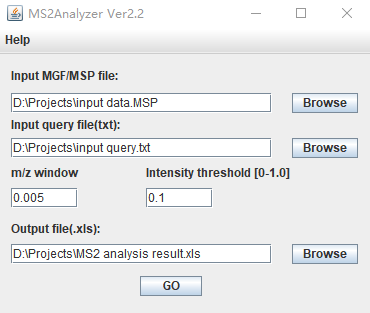
Basic parameters of MS2Analyzer. The input file, containing the raw mass spectrometry data, should be in MSP or MGF format. The query file inputs the mass spectrometry characteristic information to be searched for. The m/z window is adjusted according to the resolution of the mass spectrometry instrument. The intensity threshold, based on the relative intensity of the base peak, is used to screen out interfering peaks.

Neutral loss groups were annotated using the MS2Analyzer software. Specifically, the mass spectrometry data of the metabolic signals (including precursor ions, product ions, retention time, and other detailed spectral information) were imported into MS2Analyzer in MGF file format. MGF files can be directly exported from raw data files using manufacturer-provided software (e.g., Agilent Mass Hunter) or converted using ProteoWizard software. Query files containing precursor ions, product ions, and neutral loss information were imported in TXT format. The neutral loss information can be obtained through literature search [8] and public mass spectrometry databases (e.g., NIST, MassBank, METLIN). The basic parameters were then set, with a mass-to-charge ratio deviation range of 0.005 and a peak response intensity threshold of 0.1 (Figure 4).

**Database matching and metabolite identification**

**Figure 5.**
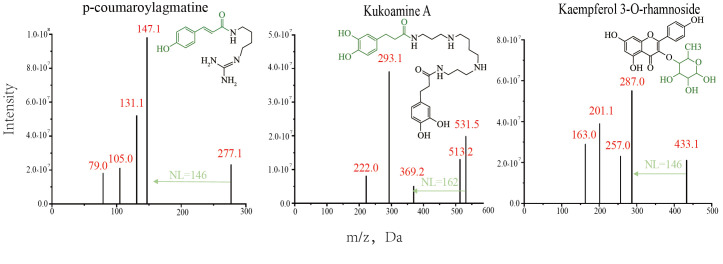
Structures and MS2 spectra of the modified metabolites. The green arrows indicate the one-step neutral loss present in the MS2 spectrum of the modified metabolite, corresponding to the green modified group in the compound structure.

In order to improve the efficiency of metabolite identification, the obtained m/z, RTs, fragmentation patterns, and neutral losses were first compared with an internal standard compound library. For example, in the identification process of the compound NL08700 (Figure 3), its m/z = 277.1 Da, RT = 2.50, NL = 146 Da, and fragmentation pattern parameters match that of the standard compound p-Coumaroylagmatine (Figure 5), thus it was annotated as p-Coumaroylagmatine. For metabolites lacking standard references, after systematically summarizing the elution patterns, fragmentation patterns, and other information from reported literature, online databases, and self-built laboratory metabolite databases, they were identified through manual spectrum interpretation. Taking the identification of the compound NL17813 as an example, its metabolic signal's RT is 4.23 min, indicating it is a moderately polar compound, and its MS2 information shows a characteristic peak of kaempferol at 287 Da. The structure is presumed to be a flavonoid with kaempferol as the parent nucleus. Further analysis of neutral losses revealed a fixed mass difference of 146 Da in the MS2 information, which may correspond to a neutral loss of rhamnose. Combining the fragmentation pattern (sugar loss in one step) and the m/z of 433.1 Da, the structure was confirmed as kaempferol 3-*O*-rhamnoside (Figure 5). Additionally, high-resolution raw data (RAW format) were submitted to Compound Discoverer 3.1 for database matching and compound annotation, which further aids in manual spectrum interpretation.

**Metabolite annotation with MetDNA**

The process of metabolite annotation using MetDNA involves the following steps: First, the known metabolite information is converted into MS1 peak tables (CSV format) and MS2 files (MGF or MSP format) using software such as ProteoWizard or MS-DIAL, ensuring the inclusion of mass-to-charge ratios, retention times, and corresponding MS2 fragment information. These data files are then imported into MetDNA, where known metabolites are used as seed metabolites. Based on the fragmentation information of the seed metabolites and their association with the Metabolic Reaction Network (MRN), neighboring metabolites on the same metabolic pathway are iteratively annotated, expanding the scope of annotation. Finally, annotation confidence is assessed based on a scoring system, redundant annotations are removed, and high-confidence metabolite annotation results are output.

**Metabolite identification levels**

The identified metabolite signals were classified into three levels of identification:

Level 1 identification: Achieved by matching the MS, MS/MS, and retention time data with reference standards.

Level 2 identification: Achieved by manual interpretation of the spectra or comparing MS, MS/MS, and retention time information with published literature and database entries.

**Figure 6.**
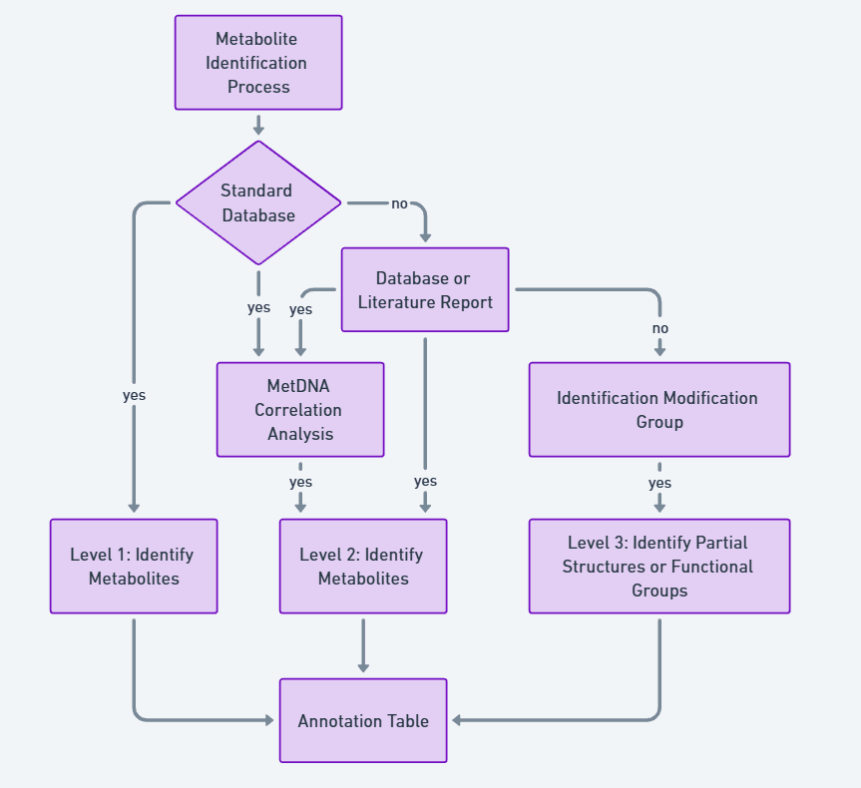
Flowchart for the grade evaluation of metabolite identification.

Level 3 identification: Involved using high-resolution parent ion data and incomplete MS/MS information to identify partial structures or functional groups (Figure 6).

**Metabolite signal optimization**

**Figure 7.**
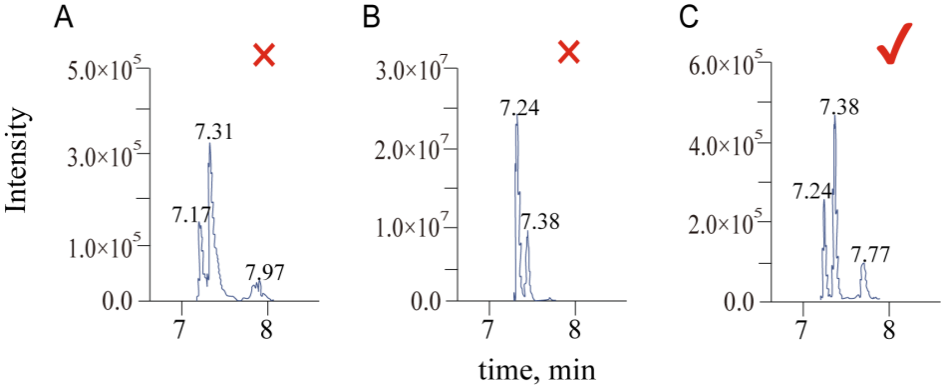
Criteria for screening chromatographic peaks. (A) Exclusion, convolution of peaks. (B) Exclusion, the relative intensity exceeding the threshold. (C) Pass, single peaks without convolution and intensities between 1×10^5^ and 1×10^7^.

For quantitative analysis using MultiQuant 3.0.3, data collected in sMRM mode was imported into the software, where a quantitative method was created. The built-in SignalFinder algorithm was used for peak integration. After integration, peaks generated from different ion pairs were screened based on their peak shape and intensity. The screening criteria included the following: chromatographic peaks should be singular (without convolution or overlap), peaks should be symmetrical, and signal intensity should range between 1×10^5^ and 1×10^7^ (Figure 7). The optimized ion pairs were then added to the database for subsequent analysis.

## Validation of protocol

This protocol or parts of it has been used and validated in the following research article:

- Yang et al. [6]. A widely targeted metabolite modificomics strategy for modified metabolites identification in tomato. JIPB.

## General notes and troubleshooting

**General notes**

1. QC data can be used for quickly confirming the stability of instrument detection and the reliability of the data. If there is a significant variation in QC data (such as signal intensity or RT) during the analysis (CV > 20%), the sample data for that batch should be considered invalid.

2. Each dataset should include at least three biological or technical replicates to ensure the reproducibility and consistency of the metabolic signals.

3. To maintain sample stability during the analysis process, it is recommended that the detection time for each batch of samples does not exceed 48 h [10].

**Troubleshooting**

**Problem 1:** Instability of column pressure.

**Possible cause:** There are many potential causes for unstable column pressure. These should be addressed one by one. If the column pressure increases, it could be due to column or tubing blockage. If the pressure decreases, it may indicate a leak in the chromatographic system connections. If the pressure fluctuates erratically, air bubbles may be present in the flow path.

**Solution:** Check whether the connections in the chromatographic system are loose. Wash or backflush the column with a gradient of 10% flow rate change, flushing for 10 column volumes per gradient ratio.

**Problem 2:** Decreased signal intensity and increased background noise.

**Possible cause:** When the ion source accumulates matrix residues from the sample, ionization efficiency can decrease significantly, leading to fluctuations or even reductions in signal intensity. Sample degradation is also a key factor affecting detection results. If samples are not stored or handled under proper temperature or light conditions, they may undergo chemical degradation, which can lead to reduced concentration and further decrease signal intensity. Insufficient vortexing can cause uneven samples, incomplete dissolution of target substances, or increased matrix effects, thus affecting the accuracy and reproducibility of the results.

**Solution:** Regularly clean the ion source. To prevent sample degradation, store samples at 4 °C for short-term storage and at -80 °C for long-term storage. For light-sensitive samples, use brown vials and store them in the dark. Ensure thorough vortexing before injection.
